# Awareness of pre-exposure prophylaxis (PrEP) among women who inject drugs in NYC: the importance of networks and syringe exchange programs for HIV prevention

**DOI:** 10.1186/s12954-017-0166-x

**Published:** 2017-06-29

**Authors:** Suzan M. Walters, Kathleen H. Reilly, Alan Neaigus, Sarah Braunstein

**Affiliations:** 1 Department of Sociology, State University of New York at Stony Brook, Stony Brook, NY USA; 2 New York City Department of Health and Mental Hygiene, New York City, NY USA; 3 Department of Epidemiology Joseph L. Mailman School of Public Health, Columbia University, New York, NY USA; 4 Stony Brook, New York, NY 11794-4356 USA

**Keywords:** HIV, Pre-exposure prophylaxis, Sex work, Syringe exchange programs, Gender, Injection drug use

## Abstract

**Background:**

Women who inject drugs (WWID) are at heightened risk for HIV due to biological, behavioral, and structural factors. Pre-exposure prophylaxis (PrEP) could aid in HIV prevention for WWID. However, little is known about WWID awareness of PrEP, which is a necessary step that must occur before PrEP uptake. We report factors associated with greater awareness among WWID to identify efficient means of awareness dissemination.

**Methods:**

Data from the 2015 National HIV Behavioral Surveillance (NHBS) system cycle on injection drug use collected in New York City (NYC) were used. Bivariable analyses, using chi-squared statistics, were conducted to examine correlates of awareness of PrEP with socio-demographic, behavioral, and health care variables. Multivariable logistic regression was used to estimate adjusted associations and determine differences in awareness of PrEP.

**Results:**

The analysis consisted of 118 WWID. Awareness of PrEP was relatively low (31%), and risk factors were high. In the last 12 months, almost two thirds (65%) reported condomless sex, approximately one third (31%) reported transactional sex, and one third (32%) reported sharing injection equipment. In multivariable logistic regression, increased PrEP awareness was associated with reported transactional sex (AOR 3.32, 95% CI 1.22–9.00) and having a conversation about HIV prevention at a syringe exchange program (SEP) (AOR 7.61, 95% CI 2.65–21.84). We did not find race, education, household income, age, binge drinking, or sexual identity to be significantly associated with PrEP awareness.

**Conclusions:**

Large proportions of WWID were unaware of PrEP. These findings suggest that social networks (specifically sex work and SEP networks) are an efficient means for disseminating messaging about prevention materials such as PrEP. We recommend that SEP access increase, SEP processes be adopted in other health care settings, and WWID networks be utilized to increase PrEP awareness.

## Background

People who inject drugs (PWID) are at increased risk for HIV infection. In 2015, injection drug use accounted for 2392 (6%) of the 39,513 estimated new HIV diagnoses in the USA. Although this may seem low, HIV nonetheless disproportionately impacts PWID [1]. It is estimated that the proportion of people who have injected drugs in their lifetime in the USA is 2.6% (3.6% for males and 1.6% for females) [2]. Yet, PWID account for doubling these proportions in new HIV diagnoses. Women who inject drugs (WWID) experience an even greater burden of HIV than men who inject [3–6]. Structural and contextual factors such as poverty, race/ethnicity, incarceration, violence, abuse, gender inequalities, marriage, discrimination, marginalization, and powerlessness can contribute greatly to a woman’s ability to prevent HIV on an individual level [7–14]. This is because life circumstances impact behavioral decisions, such as the ability/power to negotiate condom use [15–17]. Indeed, research has shown that WWID, compared to males who inject drugs, have increased injection and sexual risk behaviors [18, 4, 19–21]. In fact, it is estimated that 1 out of 23 WWID will be diagnosed with HIV in their lifetime [22]. Recent outbreaks of HIV attributed to needle and injection equipment sharing, such as the outbreak in Scott County, Indiana, serve as a warning that HIV prevalence among PWID could rapidly increase in the future. Further, PWID may serve as a bridge to other populations, creating greater HIV risk for non-PWID [23].

In terms of sexual risk for WWID, a specific sexual risk behavior linked to higher risk for HIV is transactional sex (i.e., selling sex for money or drugs) [24–26]. Studies have shown that among WWID, those who sell sex are at higher risk for HIV compared to those who do not [27, 28]. Furthermore, WWID engage in transactional sex more frequently than men who inject drugs [29]. Research demonstrates that WWID, who engage in transactional sex, have increased risk for HIV due to structural factors that limit their ability to make choices and negotiate condom use [15, 30–33]. Thus, HIV risk associated with transactional sex is not transactional sex itself, it is transactional sex without a condom or other factors that can expose an individual to HIV. Some research shows that women who engage in transactional sex are well networked, which can increase their agency to make choices and their ability to protect themselves [34–38]. This is because networks play a critical role in influencing individual behaviors, as they can increase and/or decrease risk behaviors [39].

Aside from sexual and injection risk, PWID face further disparities due to socio-demographic factors. For instance, Black and Hispanic PWID have higher prevalence of HIV [40–42]. In addition, poverty, homelessness, binge drinking, non-injection drug use (particularly crack cocaine), and incarceration have been associated with greater HIV risk behaviors among PWID [4, 43–50]. This demonstrates the importance of examining groups at risk, such as PWID, along the lines of intersecting identities (i.e., race, gender, and class) for targeted prevention efforts [51–53]. Further, it highlights the need for understanding social structures, such as networks and group dynamics for disseminating prevention materials.

### PrEP

Pre-exposure prophylaxis (PrEP) is a biomedical intervention (a pill that is taken daily to prevent HIV infection), which provides additional HIV prevention options beyond traditional modalities (i.e., condoms) for individuals at risk [54–57]. Although most of the focus around PrEP has been administering the drug to men who have sex with men (MSM), PrEP has been deemed an effective tool for preventing HIV among PWID [55]. In 2013, the Centers for Disease Control (CDC) released guidelines for administering PrEP to injection drug users (i.e., PWID) [58]. Later, in 2015, a report by the CDC estimated that 18.5% of PWID had indications for PrEP [59]. The estimate did not include sexual risk, which research shows is an important factor to consider for PWID. We, therefore, can assume that the percentage for PWID who would meet the CDC guidelines for indications for PrEP are higher. This underscores the importance of PrEP for PWID when other interventions (such as condoms and clean needles) may not be working.

A critical step in accessing PrEP for individuals at risk is awareness that PrEP exists. Studies of PrEP awareness tend to focus solely on men who have sex with men (MSM), finding low awareness among MSM [60–64], and even lower awareness among Black MSM [65]. However, in NYC, awareness among MSM was found to be higher [66]. Other studies demonstrate even lower PrEP awareness among women [67–69].

Unfortunately, little is known about PrEP awareness among PWID [70], and even less among WWID [5]. From the studies that have been conducted, awareness of PrEP among PWID has been reported much lower than the MSM population [71–73]. This is troubling since awareness of PrEP is an important step to PrEP uptake, as people need to be aware and informed about the drug before taking it.

PrEP may be particularly important for WWID because it has the capacity to increase agency and control in their lives [74]. For example, in situations where women are unable to negotiate condoms because of unequal power dynamics, PrEP could prevent HIV infection. Therefore, PrEP can provide further protections when other interventions (such as condoms) fail. However, a necessary step for PrEP uptake is awareness that the drug exists. Therefore, examining PrEP awareness is critical for public health so that areas for interventions can be identified and awareness rates can be improved.

In this paper, we examine awareness of PrEP among WWID, who were sampled in New York City as part of the National HIV Behavioral Surveillance system. We first examine risk behaviors among WWID in relation to PrEP awareness, thus demonstrating a need for HIV prevention (including PrEP) among WWID. The behaviors we examine include injection and sexual risk (including transactional sex) homelessness, incarceration, and binge drinking. We then use multivariable logistic regression to identify correlates of increased PrEP awareness to inform the development of prevention strategies for WWID.

## Methods

This analysis utilized data collected by the National HIV Behavioral Surveillance (NHBS) system, a cross-sectional survey that rotates annually among three at-risk populations for HIV infection in 22 USA metropolitan statistical areas (MSAs) with high HIV prevalence. Study protocols and questionnaires were distributed by the Centers for Disease Control and Prevention (CDC). Data used for this analysis were from the NYC fourth round of the injection drug use (IDU) cycle collected in 2015. Data were collected using respondent-driven sampling (RDS), a peer driven, chain referral sampling method that is used to survey hidden populations [75]. The NHBS protocol, methodology, and questionnaire were reviewed and approved by the NYC Department of Health and Mental Hygiene Institutional Review Board.

Prior to data collection, formative research was conducted to inform study implementation, including recommendations for survey incentives and selecting seeds (initial recruits) for RDS sampling [76]. NHBS eligibility requirements included being 18 years of age or older, being able to respond to the survey in English or Spanish, having a valid coupon for RDS sampling, residing in NYC, and having injected drugs without a prescription within the 12 months before the interview. Additional seed criteria required that the participant be either male or female, not transgender. Transgender persons were eligible for the survey, but they were excluded from this analysis due to small numbers. Anonymous and incentivized interviews were conducted face-to-face, using CDC-provided standard NHBS questionnaires. Consenting and eligible participants were interviewed and offered anonymous HIV testing, with test results returned after the survey. The results were recorded and linked to the survey responses for each participant.

### Measures

#### Dependent variable

For this analysis, the outcome of interest was awareness of PrEP. Participants who self-reported HIV-negative status were asked: “Before today, have you ever heard of people who do not have HIV taking PrEP, the antiretroviral medicine taken every day for months or years to reduce the risk of getting HIV?” Response options were yes or no.

#### Independent variables

##### Socio-demographics

Socio-demographic controls for age, education, household income, sexual identity, and mutually exclusive race/ethnicity were included. The racial/ethnic categories used in this analysis were Hispanic/Latina, African American/Black, and a category including white, multiracial, and other. If a person indicated Hispanic or Latina heritage, they were coded as Hispanic/Latina. The “multiracial/other” category included one woman who identified as Alaska Native or American Indian and one woman who identified as multiracial. Due to the small sample size of the multiracial/other category, these two WWID were combined with White for the analysis.^1^ Analyses were ran excluding the multiracial/other category (not shown), and the results were the same. Sexual identity was defined as identifying as either heterosexual or “straight,” or homosexual, lesbian, gay, or bisexual (LGB).

##### Transactional sex

Transactional sex was defined as receiving money or drugs in exchange for sex within the last 12 months from one or more sex partners. Sex was defined as vaginal, oral, or anal sex with a male partner.

##### Syringe exchange prevention conversation

Access to HIV prevention at an SEP was assessed through the following questions: “In the past 12 months, have you had a one-on-one conversation with an outreach worker, counselor, or prevention program worker or participated in an organized group session to discuss ways to prevent HIV infections?” If the participant answered yes, they were asked a series of questions as to where the conversation could have occurred, with the following response options: HIV/AIDS focused organization; gay, lesbian, bisexual, transgender, or queer organization; needle or syringe exchange program; IDU outreach program; doctor’s office, health center, clinic, or hospital; drug or alcohol treatment center; or other community organization. Binary variables were created for all options and were tested in the bivariable and multivariable models.

##### Binge drinking

Binge drinking was defined as having four or more drinks “in about 2 h” within the last thirty days.

##### Bivariable analyses

In bivariable analyses, additional variables were assessed in relation to awareness of PrEP. These included homelessness, which measured whether a participant reported being homeless (living on the street, in a shelter, in a single room occupancy hotel, or in a car) in the last 12 months. To examine whether access to health care systems yielded greater PrEP awareness, the following variables were tested during bivariable analysis. First, to measure current health care status participants were asked “Do you currently have health insurance or health care coverage?” Second, participants were asked “Is there a place that you usually go when you are sick or you need advice about your health? Do NOT include internet web sites,” measuring if they have a usual source of care. Third, whether WWID had seen a health care provider within the last 12 months was assessed through the following question: “In the past 12 months have you seen a doctor, nurse, or other health care provider?” Last, the binary variable measuring having an HIV prevention conversation at a "doctor’s office, health center, clinic, or hospital" was tested.

Additional risk factors examined in bivariable analysis were whether a participant had condomless anal or vaginal sex within the last 12 months (participants were asked a series of questions about whether they had vaginal or anal sex with reported casual and main partners without a condom within the last 12 months. If a participant reported having condomless sex for any of these questions they were coded as having condomless sex within the last 12 months.), shared injection equipment and/or needles/syringes in the last 12 months, received an STI test within the last 12 months, had ever been diagnosed with hepatitis C(HCV), and had ever been incarcerated.

#### Analysis

The analysis sought to explore what factors were associated with greater awareness of PrEP among WWID. We limited the analysis to WWID who had a completed, valid survey and answered questions associated with the variables included in this analysis. First, descriptive statistics were calculated for socio-demographic, health care and prevention, and behavioral risk factors. Second, bivariable analyses, using chi-squared statistics, were conducted to examine correlates of awareness of PrEP with identified variables. Health care, prevention, and behavioral variables that were not significant at the *p* < .20 level were excluded from the multivariable analysis. For the multivariable analysis, we added variables one by one to create the multivariable model.^2^ Non-significant variables other than socio-demographic variables were removed from the final model; all socio-demographic variables were retained in the model regardless of statistical significance.^3^ Analyses were conducted using SAS 9.4 (Cary, N.C.).

Multicollinearity was assessed through correlation matrices and variance inflation scores (VIF). VIF scores did not exceed a value of 2.5 for any of our variables, indicating no potential problems with multicollinearity [77]. Goodness of fit was assessed by examining log likelihood statistics and through the Hosmer-Lemeshow test [78, 79]. Last, outliers and influential cases were assessed by examining plots of residuals, leverage, influence on parameter estimates, influence on model fit, influence on the estimate for each variable, and deletion differences by predicted probabilities [77]. Cases that were identified as potential outliers or influential cases were removed individually, the regression was re-run, and the results were compared to the original logit. If there were changes in the results and the model improved the log likelihood when the case was deleted, we deleted the case [77, 79]. Overall, there was one case that was deleted. When the case was deleted and the regression was run, the significance for ages 40–49 changed (lost significance) and the log likelihood (an indication of model fit) improved, thus confirming that the case was indeed influential or outlier.

## Results

A total of 518 PWID completed the survey. Of these, 372 (72%) were male and 146 (28%) were female. The sample excluded 17 WWID who self-reported HIV positive in the interview (because the question about PrEP awareness was only asked of WWID who self-reported HIV negative), and included those who answered all questions associated with the analysis, and were not deemed influential cases during model assessment. The final sample size for analysis was 118 WWID.

Table 1 provides a breakdown of demographic and behavioral variables by PrEP awareness and for the entire sample. Of the 118 WWID included in this analysis, 37 (31%) had heard of PrEP (Table 1) and only one WWID reported taking PrEP. A large portion (40%) of the women were aged 50 years or older. The racial/ethnic distribution was 38% Latina, 37% Black, and 25% White. A large portion (40%) reported having some high school or less, followed by 31% reporting high school graduate or obtaining a GED, and 29% reporting some college or above. Overall, the sample reported low annual household income: 81% reported income less than $10,000, 15% reported $10,000–$25,000, and only 4% reported $25,000 or more. Over half (58%) reported being homeless in the last 12 months. For sexual identity, 73% reported heterosexual identity and 27% reported identifying as lesbian, gay, or bisexual (LGB). Most WWID in this sample (74%) reported previous incarcerated in their lifetime.

**Table 1:** Probability of PrEP awareness by demographic and behavioral characteristics of women who inject drugs (WWID) New York City, NHBS 2015 (*n* = 118)

	Aware of PrEP (*n* = 37)		Unaware of PrEP (*n* = 81)		Total		Chi-squared statistic
Variable	*n*	%	*n*	%	*n*	Row %	
PrEP awareness	37	31	81	69	118	100	
Age							
18–29	5	38	8	62	13	100	χ^2^(3) = 1.99
30–39	7	30	16	70	23	100	
40–49	8	23	27	77	35	100	
50 and older	17	36	30	64	47	100	
Race							
Hispanic/Latina	15	33	30	67	45	100	χ^2^(2) = 0.55
African American/Black	12	27	32	73	44	100	
White/multiracial/other	10	34	19	66	29	100	
Education							
Some high school or less	13	28	34	72	47	100	χ^2^(2) = 1.09
High school graduate or GED	11	30	26	70	37	100	
Some college or above	13	38	21	62	34	100	
Household income							
Up to $10,000	32	34	63	66	95	100	χ^2^(2) = 1.24
$10,000–$25,000	4	22	14	78	18	100	
$25,000 and above	1	20	4	80	5	100	
Homelessª	22	32	46	68	68	100	χ^2^(1) = 0.07
Lesbian, gay, or bisexual identity	12	38	20	63	32	100	χ^2^(1) = 0.77
Incarceration (ever)	28	32	59	68	87	100	χ^2^(1) = 0.11
Health care/prevention							
Current health care	35	31	79	69	114	100	χ^2^(1) = 0.67
Usual source of care	35	32	75	68	110	100	χ^2^(1) = 0.16
Seen health care providerª	35	32	73	68	108	100	χ^2^(1) = 0.65
Syringe exchange prevention conversation	19	54	16	46	35	100	χ^2^(1) = 12.15***
Risk factors							
STI Testª	21	40	32	60	53	100	χ^2^(1) = 3.05^
Condomless vaginal/anal sexª	22	29	54	71	76	100	χ^2^(1) = 0.58
Transactional sexª	16	43	21	57	37	100	χ^2^(1) = 3.54^
Binge drinking^b^	13	41	19	59	32	100	χ^2^(1) = 1.75
Non-injection drug usedª	26	30	61	70	87	100	χ^2^(1) = 0.33
Injection sharingª	12	32	26	68	38	100	χ^2^(1) = 0.001
Positive HIV test	0	0	2	100	2	100	χ^2^(1) = 0.93
Hepatitis C diagnosis (Ever)	8	23	27	77	35	100	χ^2^(1) = 1.67

ªIndicates within the last 12 months

^b^Indicates within the last 30 days

^*p* < .10; ***p < .001

In terms of health care and prevention, only 4 participants (3% of the sample) reported not currently having health care. Almost half (45%) reported receiving STI testing within the last 12 months. About a third (30%) reported having a conversation about HIV prevention with someone at an SEP.

Risk behaviors/indicators were relatively high. Approximately two thirds (65%) of the sample reported vaginal and/or anal sex without a condom within the last 12 months. In terms of sharing syringes and other injecting equipment, 38 WWID (32%) reported sharing injection equipment and/or syringes within the past 12 months. Just over a quarter of WWID (27%) reported binge drinking in the last 30 days. Thirty-five (37%) WWID reported ever being diagnosed with hepatitis C and 2 (2%) tested HIV positive. Since the survey only asked those who did not report positive HIV status about their HIV status, the two cases that tested positive were unaware of their status. More troubling is that the two cases that were HIV positive were unaware of PrEP.

Transactional sex was relatively high as well, with 31% of WWID sampled reporting transactional sex in the last 12 months. To further identify the sexual and injection risks of those engaging in transactional sex (as they could be minimal or large), we stratified the data to those who engaged in transactional sex and examined risk behaviors (Table 2). Out of the 37 WWID who reported transactional sex, 29 (78%) reported condomless sex in the last 12 months, 9(24%) reported having a positive HCV test, and one (3%) tested positive for HIV.

**Table 2:** Risk factors of women who inject drugs who engaged in transactional sex, New York City, NHBS 2015 (*n* = 37)

Variable	Number	Percent
STI testª	16	43
Condomless vaginal/anal sexª	29	78
Binge drinking^b^	10	27
Non-injection drug usedª	32	86
Injection sharingª	9	24
Positive HIV test	1	3
Hepatitis C diagnosis (ever)	9	24

ªIndicates within the last 12 months

^b^Indicates within the last 30 days

In multivariable logistic regression when controlling for race, household income, education, age, sexual identity, binge drinking, transactional sex, and having had a conversation about HIV prevention at an SEP (Table 3), we find that WWID, who reported transactional sex, were over three times more likely to report awareness of PrEP compared to WWID who did not report transactional sex (AOR 3.32, 95% CI 1.22–9.00).

**Table 3:** Logistic regression examining knowledge of PrEP among women who inject drugs (WWID) in New York City: National HIV Behavioral Surveillance system injection drug user cycle, 2015 (*n* = 118)

	AOR	95% CI
Race		
Hispanic/Latina	1.19	0.35–3.97
African American/Black	0.34	0.85–1.35
White/multiracial/other	Ref	
Education		
Some high school or less	Ref	
High school graduate or GED	1.3	0.42–4.03
Some college or above	2.2	0.69–7.04
Household income		
Up to $10,000	Ref	
$10,000–$25,000	0.33	0.07–1.45
$25,000 and above	0.79	0.05–12.46
Age (years)		
18–29	0.8	0.15-4.43
30–39	0.39	0.10–1.59
40–49	0.31^	0.09–1.06
50 and older	Ref	
Transactional sexª		
Yes	3.32*	1.22–9.00
No	Ref	
Syringe exchange prevention conversation		
Yes	7.61***	2.65–21.84
No	Ref	
Lesbian, gay, or bisexual (LGB)		
Yes	1.94	0.70–5.33
No		
Binge drinking^b^		
Yes	2.63^	0.89–7.76
No		

ªIndicates within the last 12 months

^b^Indicates within the last 30 days

For a two-tailed test: ^indicates *p* < .10, *indicates *p* < .05, and ***indicates *p* < .001

Log likelihood = −117

Additional significant findings were that WWID who had a conversation about HIV prevention at an SEP were over seven and a half times more likely to be aware of PrEP (AOR 7.61, 95% CI 2.65–21.84). We did not find race, education, household income, age, binge drinking, or sexual identity to be significantly associated at the *p* < 0.05 level with awareness of PrEP. Health care variables, including variables indicating conversations about HIV prevention at other settings (such as at a doctor’s office, health center, clinic, or hospital) were tested in the multivariable model, and there were no correlations. Therefore, those variables were removed from the analysis.

## Discussion

This analysis found a higher rate of PrEP awareness (31%) among WWID than other studies have shown; however, studies examining this population are sparse. In a previous study using NHBS data that looked at PWID in 2012, Walters et al. found that awareness of PrEP and post exposure prophylaxis (PEP) for PWID in NYC was only 12% (Walters et al., forthcoming). The increased awareness in a three-year period is encouraging. Even so, more needs to be done to increase awareness among this key population at risk.

### Transactional sex

We found that WWID, who engaged in transactional sex, had higher awareness of PrEP compared to WWID who did not report transactional sex. Research indicates that transactional sex is associated with higher risk for HIV transmission [24–28]. and within this sample, we find that a large portion (78%) of WWID who engaged in transactional sex reported condomless sex. This may be because (as previous research suggests) women find condom negotiations difficult due to inequalities and unequal power dynamics [15, 30–33]. Further, out of the 27 WWID who reported transaction sex, 16 (43%) had a STI test in the past 12 months, 10 (27%) reported binge drinking in the past 30 days, 32 (86%) reported non-injection drug use in the past 12 months, 9 (24%) reported injection sharing, and 9 (24%) reported having a positive HCV test within their lifetime. These risk behaviors clearly demonstrate a need for prevention, particularly PrEP, as PrEP can provide protections when condom negations fail, among WWID who engage in transactional sex.

Fortunately, we found that WWID who engage in transactional sex have greater PrEP awareness compared to WWID who do not engage in transactional sex. The increased awareness suggests that WWID, who also engaged in transactional sex, may be educating themselves through their networks about PrEP. Research has shown sex worker networks to be organized and strong [34–36, 38, 80]. Additionally, research has demonstrated how women who sell sex, even when constrained by society and disadvantaged circumstances, found ways to assert their agency [38, 81–83]. Further, research highlights how selling sex economically benefits women, how women perceived selling sex to improve their life circumstances, and how selling sex provides women with opportunities that they would not otherwise have [81, 84–86]. Therefore, women who sell sex might be choosing to do so through a rational decision process along with having social networks to support them and aid in better health outcomes. The higher awareness among WWID who exchanged sex is an important result pointing towards possible avenues for more efficient transmission of knowledge and awareness of new treatment regimens.

Many women in NYC who engage in sex work belong to organized networks that they tap for resources [38, 87, 88]. Women in these networks are in constant communication through meetings, emails, and phone calls. Sex workers have organized to support each other as well as influence policy. A recent example of sex worker activism in NYC was the mobilized effort to combat a policy that allowed NYC police to use condoms as evidence for prostitution offenses. Authors carefully followed the organized effort lead by many sex workers and sex worker allies, including attending a lobbying event in Albany, NY, where activists met with policy makers to advocate for a bill that would ban the use of condoms as evidence. The bill passed the senate and condoms are no longer used as evidence in NYC [89]. This example demonstrates the strength of sex worker networks.

Informal networks exist as well, especially among street-based sex workers, where women work collectively to protect each other against violence and ensure positive life outcomes. Additionally, NYC provides resources for sex workers, which are potential spaces and avenues for networks to form and develop. Formative research collected in 2016 with female sex workers confirmed that women who sell sex are well networked. Networks were developed independently, but also at CBOs (especially SEPs) where women utilized resources and spaces to congregate. Formative research also unveiled a pattern of drug use among street-based sex workers, which included injection drug use (NHBS high risk women formative research report 2016, unpublished). It may be through networks that women who sell sex increase their agency and can spread information, such as PrEP awareness. Other groups have networks as well, including WWID who are not engaging in sex work. These can be utilized to promote PrEP.

### SEPs

Another key finding that supports network theory is that awareness is spreading through syringe exchange programs (SEPs) via conversations about prevention. Studies have shown that availability of SEPs reduces the amount of new HIV infections among PWID [90–92]. SEPs not only provide clean syringes, but they link PWID into additional services needed, such as drug treatment programs [93]. They also dispose of syringes, conduct HIV and HCV testing, provide condoms and other safer sex items, and refer people to housing services when needed [94].

We examine SEPs as institutions that can take form as networks and/or provide spaces for networks to develop. WWID who had a conversation about prevention at an SEP were over seven times more likely to be aware of PrEP. Of all the places in which HIV prevention conversations occurred, including health care facilities, SEPs were the only places that were associated with greater PrEP awareness in this analysis. This finding strongly points to the importance of SEPs for HIV prevention, even beyond provision of safe injecting equipment, as previous research suggests [94].

NYC differs greatly in relation to other USA cities in that SEPs are prevalent and accessible. There are currently 49 locations where PWID can access SEP services in NYC. Neighboring areas such as Newark and Long Island only have one SEP in their region to cover the entire city/location. Knowing this, it is reasonable to infer that awareness of PrEP among WWID in NYC may be higher than in communities elsewhere in the USA. Further research should examine this. Additionally, public health officials should examine how workers at SEPs are approaching PrEP with their clients so that the strategies employed can be universalized in other settings. SEPs may offer a non-stigmatizing environment in which prevention can be delivered, allowing for open and honest conversations about prevention to occur. Research shows that moralizing behaviors and identities has negative health outcomes [38, 95–99]. Part of the reason SEPs are successful for prevention is that, for the most part, they avoid moralizing PWID and offer judgment free services. Other CBOs and health care providers could learn from SEPs. When WWID are comfortable and there is good communication and trust, they will be more likely to disclose behavioral habits that could place them at risk for HIV, which could lead to prevention conversations [100]. This analysis demonstrates that prevention conversations can lead to greater awareness of PrEP, and therefore, attempts to encourage and foster these types of conversations must occur.

### Policy recommendations

NYC has launched a campaign for PrEP that includes advertisements throughout the city, including in subway trains and on buses. Currently, most of these advertisements target MSM, transgender individuals, and heterosexuals. Due to marketing targeting populations other than PWID, WWID may not identify with the advertisements and make the connection that PrEP is a pill that could benefit them. Although some WWID may identify with heterosexual advertisements, targeting WWID more specifically could be beneficial so that those who do not identify with the current advertisements can be reached. Research has shown the importance of gender specific and culturally competent prevention efforts [101]. Advertisements should follow this recommendation as well.

Additionally, relevant conversations with health care providers and members of social networks are critical for increasing PrEP awareness. Specifically, conversations among peers are more likely to increase acceptance and adaptation [102]. For increased awareness and adoption of an innovation to be achieved, people need to know others within their social networks who are using (and/or talking about) the innovation. The higher the prevalence of social network members who adopt an innovation, the more likely a network member will be to adopt (and know about) it an innovation [103, 104]. Therefore, networks and communities should be targeted and utilized to increase PrEP awareness. These interventions should consider the customs of groups, including their historical and cultural frameworks [105].

Currently in NYC, there are networks of PWID. One example is Voices of Community Activists and Leaders (VOCAL-NY) which is a “grassroots membership organization building power among low-income people affected by HIV/AIDS, the drug war and mass incarceration, along with the organizations that serve us, to create healthy and just communities.” VOCAL-NY’s current campaigns include syringe access and overdose prevention. PrEP is not one of their campaigns. Rather, VOCAL-NY recently has been at the forefront in pushing for supervised injection facilities (SIFs). In May 2017, VOCAL-NY cosponsored with the Drug Policy Alliance a (mock) pop up SIF and “Everywhere but Safe” film viewing to promote awareness and gain support for SIFs [106–108]. Two years earlier in 2015, the premier of “Everywhere but Safe” launched the campaign (Safe Injection Facilities in New York City (SIF NYC)), comprised of numerous organizations and groups, working towards implementing SIFs in NYC [109, 110]. Like VOCAL-NY, neither the Drug Policy Alliance nor SIF NYC list PrEP as a cause they are advocating for. Similarly, other groups, such as Injection Drug Users Health Alliance and Harm Reduction Coalition are pushing for SIFs and/or access to syringes, but do not mention PrEP [111, 112]. These organizations would be ideal to work with to spread PrEP awareness as they are already integrated in the community. However, these groups may not be pushing PrEP out of fear that PrEP implementation might take away from other, more immediate, harm reduction needs (such as SIFs) [113]. Public health officials should consider how to integrate PrEP within the harm reduction frameworks established.

Another potential route to increase awareness (and where conversations about PrEP should occur) is through interactions with health care providers. This study did not find an association with having a usual health care provider, receiving free condoms at a doctor’s office, health center, clinic or hospital, receiving syringes at a doctor’s office, health center, clinic or hospital, or having a prevention conversation at a doctor’s office, health center, clinic, or hospital, and awareness of PrEP for WWID. Previous research has shown that providers were more willing to prescribe PrEP to MSM and less willing to prescribe to PWID [114]. Additionally, prescribers of PrEP have reported mixed feelings about PrEP, including confliction in prescribing PrEP, due to concerns about side effects, drug effectiveness, concerns about risk disinhibition, and ideas of who appropriate patients would be [115, 116]. Therefore, it may be a lack of provider willingness to inform WWID about PrEP that explains the lack of correlation found in this study. We suggest that clinicians and other persons inside health care setting, along with service organizations outside of health care settings, be trained in PrEP. Specifically, we suggest that they are aware of the distinct needs of WWID. WWID often occupy many marginalized and stigmatized positions in society, which can create barriers to health care. Providers need to be welcoming and non-judgmental towards WWID for risk behaviors to be disclosed. Through open, non-stigmatizing interactions and dialog, greater PrEP awareness can be achieved. More research on prescriber perceptions, specifically as they relate to WWID, needs to be conducted so that efficient communication about HIV prevention can occur among health care providers and WWID. In addition, more training on PrEP and the importance of PrEP, specifically for WWID, among health care providers and other service organizations could benefit public health prevention efforts related to PrEP.

### Limitations

This study is subject to several limitations. First, the cross-sectional nature of this study precludes the determination of temporal relationships. Although one of the advantages of RDS is that it has the potential to reduce bias by producing long recruitment chains that bridge relevant groups, it is possible that this study oversampled from well-connected social networks that differ from the target population particularly with respect to housing status, income, and employment. The data is unweighted because assumptions needed for weighting were not met, and therefore is not a probability sample [117–122]. The results of this study may not be generalizable. Second, this study did not differentiate between types of transactional sex (e.g., commercial sex, survival sex). There may be differences in perceptions of what constitutes transactional sex, which could have affected women’s reporting. Third, the study questionnaire elicited sensitive information regarding sexual behavior and HIV status, and participants may not have felt comfortable disclosing this information to study staff interviewers. However, studies of PWID show high levels of reliability and validity when reporting sensitive behaviors [123]. Fourth, the question assessing awareness of PrEP was only asked to self-reported HIV-negative participants. We, therefore, were unable to test if HIV status was associated with awareness of PrEP. Last, the limited sample size may have restricted the power to detect significant differences within the target population and may have impeded the inclusion of other relevant variables in the final multivariable models or may have led to spurious associations, which would be mitigated by a larger sample. Some point estimates had wide confidence intervals reducing the precision of estimated effects.

## Conclusions

PrEP has the potential to increase agency and control with regard to HIV risk in the lives of those who take it as part of combination HIV prevention since individual-level preventions such as condoms do not always work. WWID, especially those who engage in transactional sex, could greatly benefit from PrEP. Out of the 37 WWID who reported transactional sex, 29 (78%) reported condomless sex. PrEP may be a way for WWID, particularly those who do not have the power to negotiate condom use, to protect themselves from HIV.

Unfortunately, 69% of the sample was unaware of PrEP. However, this study suggests potential routes for disseminating awareness that can be explored and implemented in different settings. One important way to increase awareness of PrEP is by tapping into organizations (and networks) that are tailored to WWID needs, such as SEPs. Since SEPs were associated with greater PrEP awareness, examining how SEPs are informing WWID about PrEP is critical for future PrEP awareness efforts. SEP strategies can be expanded to other health care settings, leading to important and successful ways of increasing PrEP awareness. By examining how SEPs are informing WWID about PrEP, public health officials can begin to integrate SEP strategies in other settings. This finding also suggests that increasing SEPs in areas may lead to increased awareness of PrEP and more HIV prevention opportunities broadly among WWID. Another method for disseminating risk reduction information, which can be utilized to distribute PrEP information, is through outreach by WWID peers or those with privileged access to WWID (i.e., tapping into their networks). Outreach targeting PWID was among the first methods used to reduce HIV risk and has been proven successful [124, 125].

Second, networks of women who engage in transactional sex and other networks containing WWID should be utilized as avenues for disseminating PrEP awareness. The results of this study among WWID show that those who reported transactional sex had greater awareness of PrEP. This may be because women who engage in transactional sex are more tightly networked, and their networks are actively discussing HIV prevention. Providing information about PrEP to networks and encouraging dialog about it could greatly increase PrEP awareness. Even more broadly, working with other WWID networks to disseminate awareness of PrEP may be a key tool for increasing awareness generally among WWID.

## Abbreviations

CDC: Centers for Disease Control and Prevention
HIV: Human immunodeficiency virus
HCV: Hepatitis c virus
LGB: Lesbian, gay, or bisexual
MSM: Men who have sex with men
NHBS: National HIV Behavioral Surveillance system
NYC: New York City
PWID: People who inject drugs
SEP: Syringe exchange program
SIF NYC: Safe injection facilities in New York city
SIF: Supervised injection facility
VOCAL-NY: Voices of community activists and leaders
WWID: Women who inject drugs

## Footnotes

1 Throughout the paper, we refer to Hispanic/Latina as Latina, African American/Black as Black, and White/multiracial/other as White.

2 A separate analysis was conducted that controlled for network size. The results remained the same, with two key relationships strengthened: having a conversation about HIV prevention at an SEP and engaging in transactional sex.

3 To test if there was a significant interaction between syringe exchange prevention conversations and transaction sex, an interaction term was tested in the final model. The interaction was not significant and was therefore excluded.
